# Association between glycated hemoglobin and lipid parameters at the time of type 2 diabetes mellitus diagnosis: evidence from a regional population

**DOI:** 10.3389/fmed.2026.1833588

**Published:** 2026-07-14

**Authors:** Raquel Sainz-Prado, Beatriz Rodríguez-Roca, Miren Idoia Pardavila-Belio, Paula Rojas-García, Félix Rivera-Sanz, Elena Andrade-Gómez

**Affiliations:** 1 Pre-Departmental Unit of Biomedical Sciences and Health Specialties, University of La Rioja, Logroño, Spain; 2 Oncology Unit, Hospital San Pedro, La Rioja Health Service, Logroño, Spain; 3 Department of Physiatry and Nursing, Faculty of Health Sciences, University of Zaragoza, Zaragoza, Spain; 4 SAPIENF (B53_23R) Research Group, Faculty of Health Sciences, University of Zaragoza, Zaragoza, Spain; 5 School of Nursing, Community, Maternity and Pediatric Nursing, University of Navarra, Pamplona, Spain; 6 Navarra Institute for Health Research (IdiSNA), Pamplona, Spain; 7 Department of Economics and Business, University of La Rioja, Logroño, Spain; 8 Data Science Unit, Fundación Rioja Salud, Logroño, Spain

**Keywords:** clinical practice, fasting plasma glucose, glycated hemoglobin, lipid profile, nursing, total cholesterol, type 2 diabetes mellitus

## Abstract

**Background and objectives:**

Type 2 diabetes mellitus (T2DM) is associated with lipid abnormalities that increase cardiovascular risk; however, evidence regarding this relationship at diagnosis is limited. This study aimed to examine the association between glycated hemoglobin (HbA1c) and lipid parameters at the time of T2DM diagnosis.

**Methods:**

A retrospective cross-sectional study included 3,501 newly diagnosed T2DM patients treated within the Riojano Health Service between 2019 and 2025. Multiple linear regression was used to assess associations between HbA1c and lipid parameters. The study was approved by the Research Ethics Committee of La Rioja (CEImLAR, PI 778).

**Results:**

HbA1c was positively associated with total cholesterol (TC) (β = 4.25, *p* < 0.001) and triglycerides (TG) (β = 15.65, *p* < 0.001) and negatively associated with high-density lipoprotein cholesterol (HDL-C) (β = −1.40, *p* < 0.001) in multiple linear regression adjusted for age, sex, and body mass index (BMI). No significant association was found between HbA1c and low-density lipoprotein cholesterol (LDL-C) (β = 1.67, *p* = 0.080).

**Conclusion:**

At diagnosis, higher HbA1c levels were independently associated with a more atherogenic lipid profile—higher TC and TG, and lower HDL-C—highlighting the importance of early metabolic assessment and comprehensive cardiovascular risk management.

## 1 Introduction

Type 2 diabetes mellitus (T2DM) is a metabolic disorder characterized by chronic hyperglycemia resulting from impaired insulin secretion, impaired insulin action, or both (1). It is one of the most prevalent chronic diseases worldwide, currently affecting more than 463 million people, with projections estimating that this number will exceed 690 million by 2045 (2).

Individuals with T2DM present a two- to fourfold higher risk of cardiovascular disease (CVD) compared with general population, positioning CVD as the principal cause of morbidity and mortality in this group (3) and a major public health burden worldwide. From a population health perspective, inadequate glycemic control represents a key modifiable determinant of CVD risk, with glycated hemoglobin (HbA1c) functioning as a standardized epidemiological indicator of long-term hyperglycemia over the preceding 2–3 months and demonstrating a robust and consisted association with cardiovascular outcomes. Emerging evidence suggests that HbA1c may also be useful for cardiovascular risk assessment even in individuals without diabetes (4–6).

Sustained hyperglycemia contributes to CVD through multiple pathophysiological mechanisms, including insulin resistance, low-grade chronic inflammation, endothelial dysfunction, and microvascular damage mediated by advanced glycation end-products (7, 8). From a public health perspective, these mechanisms underpin the high population-attributable risk of cardiovascular complications associated with T2DM, especially when hyperglycemia remains undetected or poorly controlled. T2DM commonly coexists with other metabolic disturbances such as dyslipidemia and hypertension, which cluster at the population level and synergistically amplify cardiometabolic risk (2).

The characteristic dyslipidemia of T2DM includes elevated triglycerides (TG), increased small dense LDL particles, and reduced HDL cholesterol (HDL-C), forming a highly atherogenic profile (9, 10). These lipid abnormalities are highly prevalent in real-world clinical populations and represent a key target for early cardiovascular risk reduction strategies. Hyperglycemia and insulin resistance modify lipid metabolism and promote the accumulation of atherogenic lipoproteins, contributing to the early development of subclinical atherosclerosis (11).

Early identification and management of cardiometabolic risk factors at the time of T2DM diagnosis are essential for preventing long-term cardiovascular complications (12). Nurses play a key role in the monitoring of glycemic and lipid parameters, cardiovascular risk assessment, and patient education aimed at improving metabolic control and treatment adherence (13–15). Due to their continuous close contact with patients, nurses are strategically positioned to support early preventive measures and integrated T2DM management from the beginning of the diagnostic process (12, 15, 16).

Despite the high prevalence of T2DM and its strong association with CVD, evidence from our context is limited regarding the metabolic profile of newly diagnosed patients, and in particular the relationship between early glycemic control and lipid alterations in real-world clinical practice. Understanding this interaction at the initial stages of the disease trajectory is crucial for informing therapeutic strategies and reducing future cardiovascular risk.

In this context, population-based regional data derived from integrated healthcare systems provide valuable real-world evidence, as they reflect routine clinical practice and allow characterization of cardiometabolic profiles at the time of diagnosis in unselected populations. Therefore, this study aimed to explore the relationship between HbA1c levels and lipid parameters in adults newly diagnosed with T2DM, providing evidence to support early, prevention-oriented clinical decision making at the time of diagnosis.

## 2 Materials and methods

### 2.1 Study design

A retrospective cross-sectional study was conducted using routinely collected clinical and biochemical data from patients newly diagnosed with type 2 diabetes mellitus (T2DM). Baseline information corresponding to the time of diagnosis was obtained from electronic medical records of the Riojano Health Service (Servicio Riojano de Salud, SERIS), accessed through the Data Science, Big Data and Artificial Intelligence Unit (UCIDA) of Fundación Rioja Salud. The study included diagnoses recorded between 2019 and 2025.

### 2.2 Population

The study included 3,501 individuals with a diagnosis of T2DM registered in SERIS. Eligible participants were identified using the International Classification Diseases, 10th revision (ICD-10) Code E11 (17). Newly diagnosed T2DM was defined as the first recorded diagnosis of T2DM in the electronic health record during the study period 2019–2025, supported by standard laboratory criteria in accordance with current diagnostic guidelines. The final sample size was determined by the availability of medical records meeting the predefined inclusion and exclusion criteria during the study period.

### 2.3 Inclusion criteria

• Residence in the Autonomous Community of La Rioja.
• Diagnosis of T2DM based on ICD-10 code E11, confirmed by at least one of the following criteria:
  o HbA1c ≥6.5% (48 mmol/mol) in two separate tests
  o Fasting plasma glucose ≥126 mg/dL in two separate tests
• Age ≥ 18 years

### 2.4 Exclusion criteria (ICD-10)

Participants with conditions associated with systemic metabolic or cardiovascular impairment were excluded:

- Pregnancy
- Ongoing cancer treatment
- Alzheimer’s disease, dementia, or severe cognitive impairment
- Heart failure [New York Heart Association (NYHA) class III–IV]
- Left ventricular ejection fraction (LVEF) < 40%
- Severe functional limitations due to peripheral arterial disease

### 2.5 Data sources and data collection

Data were obtained from the Data Science, Big Data and Artificial Intelligence Unit (UCIDA) of Fundación Rioja Salud, which works with routinely collected electronic health records from the public healthcare system of La Rioja. These data derive from the entire regional healthcare network, including both primary and specialized care, and are not sourced from a single hospital or clinic. Data were not sourced from a publicly available registry and were accessed under institutional authorization. The study included data recorded between 2019 and 2025.

#### 2.5.1 Study variables

All sociodemographic, clinical, and biochemical variables were obtained from patients’ medical records. Sociodemographic variables included sex, date of birth, age, country of origin and rural/urban residence.

Clinical variables included date of diabetes diagnosis, body mass index (BMI; kg/m^2^), systolic blood pressure (SBP), and diastolic blood pressure (DBP). Clinical measurements were collected from forms within a maximum of 60 days from the diagnosis date.

Biochemical variables included glucose, total cholesterol (TC), low-density lipoprotein cholesterol (LDL-C), high-density lipoprotein cholesterol (HDL-C), triglycerides (TG) and glycated hemoglobin (HbA1c). Laboratory values were obtained under routine fasting conditions, according to standard clinical practice, and corresponded to the measurement closest to the diagnosis date within a maximum window of 60 days.

### 2.6 Ethical considerations

The study was conducted in accordance with the principles of the Declaration of Helsinki and applicable national and institutional regulations on human subject research. Informed consent from patients was not required, as only fully anonymized data from medical records were used.

Data were provided by the Data Science, Big Data, and Artificial Intelligence Unit (UCIDA) of the public health system of La Rioja, within the framework of a project approved by the Research Ethics Committee of La Rioja (CEImLAR, PI 778, September 2025). Patient data were coded and handled in compliance with current legislation on data protection and used exclusively for research purposes.

### 2.7 Statistical analysis

Statistical analyses were conducted using IBM SPSS Statistics version 28.01.1 (18). Categorical variables were described as frequencies and percentages (n, %); continuous variables were summarized as mean ± standard deviation (SD).

The distribution of continuous variables was evaluated using the Shapiro–Wilk test together with graphical methods (histograms and Q–Q plots). Differences in lipid parameters across clinically relevant HbA1c categories were assessed using one-way analysis of variance (ANOVA) for normally distributed variables (TC, LDL-C, and HDL-C), and the Kruskal–Wallis test for TG, which showed a non-normal distribution.

Associations between HbA1c and lipid parameters were initially explored using Spearman correlation coefficients. To account for potential confounding, multiple linear regression models were performed using HbA1c as the main predictor of lipid profile variables, adjusting for age, sex, and BMI, selected *a priori* based on their well-established clinical and epidemiological associations.

Statistical significance was set at *p* < 0.05, with a 95% confidence level.

## 3 Results

**FIGURE 1:**
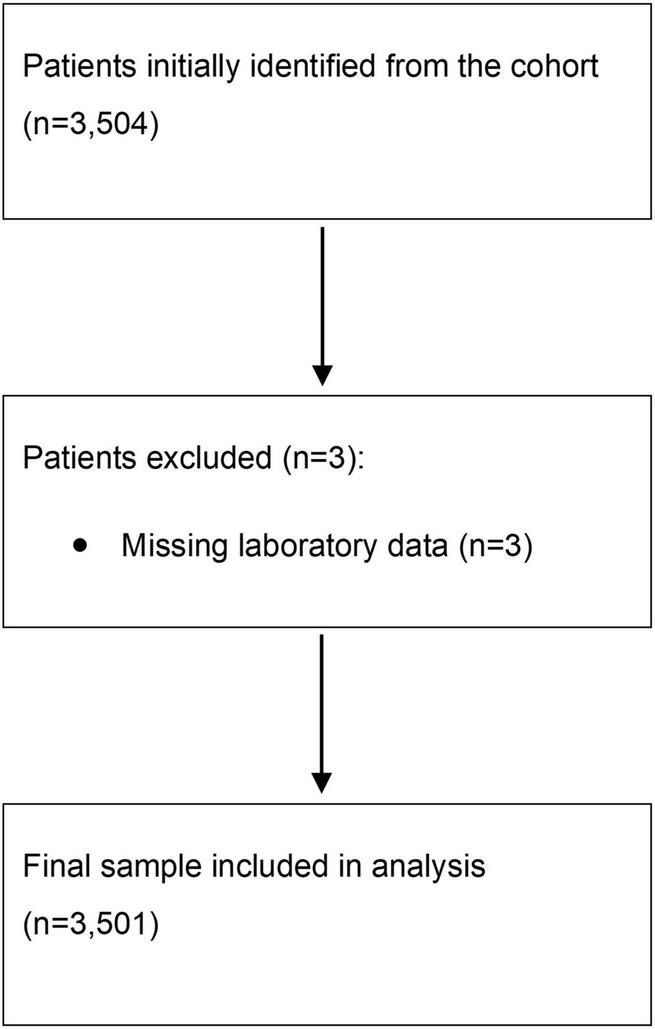
Flow diagram of participant selection for the cross-sectional analysis.

A total of 3,504 patients were initially identified. Three patients were excluded because no laboratory information was available at diagnosis, resulting in a final sample of 3,501 patients included in the analysis (Figure 1).

Not all variables were available for every participant; therefore, an available-case analysis approach was used. Consequently, the number of observations varied across variables according to data availability. Participants with missing data for a given variable were excluded only from analyses involving that specific parameter. Extreme or implausible values were excluded when identified.

### 3.1 General characteristics

The study included 3,501 patients with a mean age of 67.9 ± 12.94 years; 56.2% were men and 43.8% were women. The majority (84.6%) were from Spain, whereas 15.4% were from other countries. Most patients (67%) lived in urban areas, while 33% resided in rural settings.

Regarding the distribution of diagnoses over time, 15.1% of patients were diagnosed with T2DM in 2019, 14.3% in 2020, 15.9% in 2021, 17.8% in 2022, and 17.1% in 2023. A marked decrease was observed in 2024, which persisted into 2025, with 9.9% of diagnoses occurring in each year.

### 3.2 Clinical and biochemical parameters

Table 1 presents the mean values, standard deviations, and number of valid observations for the main clinical and biochemical variables, including glucose, HbA1c, lipid profile, BMI, and blood pressure, stratified by sex and for the total population. Standard reference ranges were based on widely accepted clinical guidelines and aligned with institutional laboratory standards (1, 19–21).

**TABLE 1:** Clinical and biochemical parameters in men, women, and the total population.

Variable	Total mean ± SD (N)	Men mean ± SD (N)	Women mean ± SD (*N*)	Standard ranges
Glucose (mg/dL)	157.19 ± 53.82 (3,308)	159.98 ± 55.83 (1,865)	153.58 ± 50.90 (1,443)	70 – 100
HbA1c (%)	7.3 ± 1.55 (56 mmol/mol) (2,898)	7.4 ± 1.63 (57 mmol/mol) (1,630)	7.2 ± 1.45 (55 mmol/mol) (1,268)	<5.7
TC (mg/dL)	191.93 ± 43.73 (3,107)	187.17 ± 44.72 (1,757)	198.12 ± 41.61 (1,350)	<200
LDL-C (mg/dL)	109.49 ± 36.02 (2,728)	107.18 ± 36.40 (1,517)	112.37 ± 35.33 (1,211)	<100
HDL-C (mg/dL)	48.32 ± 12.82 (2,995)	45.47 ± 11.87 (1,697)	52.03 ± 13.08 (1,298)	Men ≥ 40 Women ≥ 50
TG (mg/dL)	175.84 ± 105.59 (3,010)	180.06 ± 117.33 (1,702)	170.35 ± 87.75 (1308)	<150
BMI (kg/m^2^)	31.84 ± 5.35 (827)	31.65 ± 4.98 (471)	32.08 ± 5.81 (356)	18.5 – 24.9
SBP (mmHg)	135.29 ± 16.45 (1,299)	136.53 ± 16.68 (716)	134.07 ± 16.08 (583)	<120
DBP (mmHg)	78.70 ± 10.45 (1,276)	79.96 ± 10.75 (700)	77.17 ± 9.88 (576)	<80

HbA1c, glycated hemoglobin; TC, total cholesterol; LDL-C, low-density lipoprotein cholesterol; HDL-C, high-density lipoprotein cholesterol; TG, triglycerides; BMI, body mass index; SBP, systolic blood pressure; DBP, diastolic blood pressure.

Among the 2,903 patients with available HbA1c at diagnosis, 26.4% had values between 7.0% (53 mmol/mol) and 8.9% (74 mmol/mol), and 12.4% had HbA1c ≥ 9% (75 mmol/mol). TG data were available for 1,508 patients at diagnosis. Among these, 668 patients (44.3%) had TG levels between 150 and 200 mg/dL and 840 patients (55.7%) had values >200 mg/dL.

### 3.3 Lipid profile according to HbA1c categories

When lipid parameters were analyzed according to clinically relevant HbA1c categories (Table 2), a progressive worsening of the lipid profile was observed with increasing HbA1c levels. Higher HbA1c categories were associated with higher TC, LDL-C and TG levels, together with lower HDL-C values. These differences remained statistically significant across HbA1c groups for TC, HDL-C and TG.

**TABLE 2:** Lipid profile according to clinically relevant HbA1c categories.

HbA1c category	Total cholesterol (mg/dL) mean ± SD (*N*)	LDL-C (mg/dL) mean ± SD (*N*)	HDL-C (mg/dL) mean ± SD (*N*)	Triglycerides (mg/dL) mean ± SD (*N*)
<7.0%	188.74 ± 41.34 (1,656)	107.68 ± 35.21 (1,531)	50.55 ± 12.70 (1,606)	156.18 ± 86.99 (1,610)
7.0**%**–8.9%	191.53 ± 43.77 (729)	108.95 ± 36.01 (629)	45.68 ± 12.28 (709)	188.96 ± 104.49 (711)
≥9.0%	204.30 ± 47.52 (351)	116.49 ± 37.64 (269)	42.63 ± 11.08 (342)	230.70 ± 139.71 (335)
*p*-value	<0.001	0.264	<0.001	<0.001

Differences across HbA1c categories were tested with one-way ANOVA for total cholesterol, LDL and HDL cholesterol, and the Kruskal–Wallis test for TG.

### 3.4 Correlation and regression analysis

Spearman correlation analyses showed a statistically significant but weak positive association between HbA1c and TC (ρ = 0.043; *p* < 0.05), LDL-C (ρ = 0.044; *p* < 0.05) and TG (ρ = 0.217; *p* < 0.001), as well as a negative association with HDL-C (ρ = −0.213; *p* < 0.001).

To account for potential confounding and assess independent associations, multiple linear regression analyses adjusted for age, sex, and BMI were performed. HbA1c was independently associated with higher TC and TG levels, as well as a lower HDL-C (all *p* < 0.001), whereas its association with LDL-C did not reach statistical significance.

Sex consistently demonstrated an influence across models, with women presenting higher TC, LDL-C and HDL-C levels compared with men. Age was negatively associated with TC, LDL-C and TG, while BMI showed a significant inverse association with HDL-C and a positive association with TG.

These findings indicate that, at the biochemical evaluation performed at the time of T2DM diagnosis, higher HbA1c levels are independently associated with lipid parameters, after adjustment for age, sex and BMI (Table 3).

**TABLE 3:** Effect of HbA1c, sex, age, and BMI on lipid parameters: adjusted multiple linear regression results.

Dependent variable	R^2^	Predictor	B	*p*
TC	0.106	HbA1c	4.25	<0.001
		Sex	19.76	<0.001
		Age	−0.55	<0.001
		BMI	−0.49	0.119
LDL-C	0.057	HbA1c	1.67	0.080
		Sex	10.47	<0.001
		Age	−0.54	<0.001
		BMI	−0.41	0.139
HDL-C	0.120	HbA1c	−1.40	<0.001
		Sex	6.07	<0.001
		Age	0.06	0.090
		BMI	−0.22	0.018
TG	0.116	HbA1c	15.65	<0.001
		Sex	8.28	0.264
		Age	−1.011	0.001
		BMI	1.535	0.034

B = unstandardized coefficient; *p*-values in bold indicate statistical significance (*p* < 0.05). Sex coded as 0 = male, 1 = female.

## 4 Discussion

This study examined the relationship between glycemic control and lipid profile in a large population of patients newly diagnosed with T2DM. The main finding is that higher HbA1c levels were independently associated with a more atherogenic lipid profile at diagnosis, characterized by higher TC and TG and lower HDL-C levels, after adjustment for age, sex, and BMI.

These results suggest that dyslipidemia associated with poor glycemic control is already present at the earliest stages of T2DM, highlighting the close metabolic interaction between glucose and lipid metabolism.

This finding is particularly relevant in the context of the epidemiological and clinical characteristics of the study population. The patients had a mean age of 67.9 years, a factor closely linked to the incidence of diabetes and its complications, likely related to reduced physical activity and certain lifestyle habits (22, 23). The higher prevalence of diabetes in men (56.2%) differs from what has been reported in other population contexts (24, 25). A rise in the number of T2DM diagnoses was observed in the years immediately following the COVID-19 pandemic, likely related to lifestyle changes during lockdown including reduced physical activity, poorer dietary habits, increased consumption of alcohol and tobacco, and sleep disturbances, stress, anxiety, and depression (26–28). These factors may have contributed to increases in overweight, obesity, and metabolic disorders (29) in addition to potential metabolic alterations derived from COVID-19 infection itself (30–33).

Although the association between diabetes mellitus and cardiovascular risk is well established (34, 35), the main findings of this study indicate that this relationship is present from the early stages of the disease. Even at the time of diagnosis, poorer glycemic control—reflected by higher HbA1c levels—was independently associated with a more atherogenic lipid profile, consistent with previous studies (36). Specifically, after adjustment for age, sex and BMI, each 1% increase in HbA1c (≈11 mmol/mol) was associated with an increase of 4.25 mg/dL in TC, 15.65 mg/dL in TG, and a decrease of 1.40 mg/dL in HDL-C, reinforcing evidence that chronic hyperglycemia disrupts lipid metabolism and contributes to cardiovascular risk even before clinical complications become apparent.

Sex also had a significant influence on the lipid profile: after adjustment, women presented slightly higher TC, LDL-C and HDL-C levels, in line with previous reports (37, 38). This may be related to decreased estrogen levels after menopause, which have been associated with increases in TC and LDL-C (39).

In this study, the absence of a significant association between HbA1c and LDL-C concentration in the adjusted models does not preclude the presence of atherogenic lipid alterations. Diabetic dyslipidemia is characterized by elevated TG, reduced HDL-C, increased VLDL production, and a predominance of small dense LDL particles, whereas LDL-C concentrations may remain relatively unchanged, particularly during the early stages of T2DM (40, 41). Consequently, worsening glycemic control may be associated with qualitative changes in LDL particles and increased atherogenicity without necessarily producing substantial increases in circulating LDL-C levels, as suggested by Juhi et al., who reported an increased proportion of small dense LDL particles in patients with T2DM despite normal or near-normal LDL-C levels (42).

This interpretation is supported by the observed pattern in our population, in which mean LDL-C concentrations showed a slight progressive increase across HbA1c categories but did not reach statistical significance after adjustment.

Previous studies have reported inconsistent findings regarding the relationship between HbA1c and LDL-C. Some authors have found no significant association between HbA1c and LDL-C levels (43). In contrast, Chain et al., in a cohort of newly diagnosed patients with T2DM, reported a significant positive association between HbA1c and LDL-C (44). However, their study included a substantially smaller sample and was conducted in a population from India, whose demographic, ethnic, lifestyle, and metabolic characteristics may differ from those of the present cohort.

Furthermore, studies conducted in populations with established diabetes and different sociodemographic characteristics have also described positive correlations between both parameters (45–47). For instance, Lnu et al. observed a positive association between HbA1c and LDL-C in a younger cohort; however, differences in age distribution, ethnic background, clinical characteristics, and the lack of adjustment for important confounding factors may partly explain the discrepancy with our findings (45).

Another important finding of the study was that the patients with poor glycemic control, as indicated by HbA1c ≥7%, had significantly higher TG levels. This may be explained by insulin facilitating glucose uptake and suppressing lipolysis in adipose tissue (41, 48–50). In the presence of insulin resistance or deficiency, this response is impaired, increasing the release of free fatty acids and glycerol into circulation. These substrates are subsequently transported to the liver, where they promote hepatic synthesis of phospholipids, cholesterol, and TG, resulting in increased very-low-density lipoprotein (VLDL) production and elevated circulating TG levels (49, 50). Consistent with this mechanism, our findings show that TG levels increase progressively as HbA1c rises, indicating the early development of hypertriglyceridemia in T2DM and its contribution to the atherogenic lipid profile from disease onset.

From a clinical perspective, these findings have important implications. The positive relationship between HbA1c and lipid profile in newly diagnosed T2DM suggests that HbA1c may be considered as a biomarker not only for glycemic control but also for early cardiovascular risk detection. Elevated HbA1c levels, indicating insufficient glucose regulation, may help identify patients who are more likely to present lipid abnormalities. This underscores the need for early implementation of integrated therapeutic strategies—targeted lipid control and lifestyle interventions—that simultaneously address glucose and lipid metabolism to reduce cardiovascular risk.

In this context, healthcare professionals, particularly nursing professionals, are positioned to play a proactive role in cardiovascular risk stratification, patient education, and the implementation of individualized care plans (12–14, 16). Their involvement in structured follow-up, motivational strategies, dietary counseling, and promotion of physical activity represents an essential component of comprehensive cardiovascular risk reduction and has been shown to positively influence long-term metabolic control (14, 15).

This study has some limitations that should be considered when interpreting the results. First, due to the cross-sectional design, the findings reflect associations at a single point in time, preventing causal inferences or temporal sequencing between the variables. Second, biochemical and clinical parameters may have been influenced by unmeasured confounders, such as dietary patterns, smoking status, physical activity, stress levels, acute illness, diabetes-related factors, and use of medications that could modify metabolic outcomes. In addition, systematically available information on prior lipid-lowering therapy and previously diagnosed dyslipidemia was lacking, precluding stratified analyses or adjustment for these factors and potentially contributing to residual confounding. Third, certain biochemical markers, such as TG, may exhibit temporal variability, and reliance on a single measurement could introduce information bias and limit estimation precision. Fourth, the number of observations varied across clinical and biochemical parameters due to incomplete laboratory data, which may affect the precision of estimates and comparisons between groups. These limitations highlight the need for caution when interpreting results and suggest that longitudinal research or repeated-measure studies are warranted to confirm and expand upon these associations.

Despite these limitations, this study benefits from a large, community-based sample with a wide age range (20–102 years). The substantial sample size and strong representativeness enhance external validity and provide a robust and generalizable depiction of the sociodemographic and clinical characteristics of this population.

This study addresses an important gap in the literature by providing real-world evidence on early metabolic alterations at the time of T2DM diagnosis, contributing to the understanding of metabolic risk profiles in routine clinical practice.

While causality cannot be established, cross-sectional designs offer valuable insights into the distribution and prevalence of early metabolic alterations and serve as a solid foundation for generating hypotheses and informing future longitudinal or experimental research.

## 5 Conclusion

The findings of this study indicate that, in this predominantly older adult population of mostly Spanish origin, poorer glycemic control was independently associated with a more atherogenic lipid profile at the time of T2DM diagnosis. After adjustment for age, sex, and BMI, higher HbA1c levels were associated with increased TC and TG levels and lower HDL-C concentrations, whereas no significant association was observed with LDL-C. These findings suggest that metabolic alterations linked to cardiovascular risk are already present at the early stages of T2DM.

From a clinical perspective, the results highlight the importance of early and comprehensive cardiovascular risk assessment in newly diagnosed patients with T2DM, integrating both glycemic control and detailed lipid profiling. Early identification of unfavorable metabolic profiles may facilitate timely preventive and therapeutic interventions aimed at reducing long-term cardiovascular risk.

## Data Availability

The data analyzed in this study is subject to the following licenses/restrictions: The data supporting the findings of this study are available from the corresponding author upon reasonable request. Access to the data will be granted subject to appropriate justification and compliance with relevant ethical and legal requirements. Requests to access these datasets should be directed to BR-R, brodriguez@unizar.es.
